# Evaluating consumer self-medication practices, pharmaceutical care services, and pharmacy selection: a quantitative study

**DOI:** 10.1186/s12913-023-10471-1

**Published:** 2024-01-03

**Authors:** Lusine Nazaryan, Anush Barseghyan, Maria Rayisyan, Margarit Beglaryan, Marta Simonyan

**Affiliations:** 1 Department of Pharmaceutical Management, Yerevan State Medical University after M. Heratsi, Yerevan, Armenia; 2 Department of Regulatory Relations of Circulation of Medicines and Medical Devices, I.M. Sechenov First Moscow State Medical University, Moscow, Russian Federation

**Keywords:** Pharmaceutical care, Pharmacy employees, Algorithms, Pharmacy preference, Self-medication

## Abstract

**Background:**

The primary objectives of this study were the evaluation of consumer self-medication practices, the assessment of pharmaceutical care provided by pharmacy employees, and the analysis of consumer satisfaction with such care. The research was also aimed at examining the main criteria that consumers consider important when selecting a pharmacy in Armenia.

**Methods:**

The survey was based on an anonymous questionnaire and carried out between March 2020 and November 2021. It was aimed at providing a comprehensive assessment of pharmaceutical care services and consumer pharmacy choice by investigating two distinct groups: pharmacy consumers and pharmacy employees.

**Results:**

The research reveals that many residents in Armenia engage in self-medication without consulting professional sources, which can lead to potential risks and result in dangerous consequences. This is partly due to a lack of trust in pharmacy employees, which is primarily due to their inability to provide adequate information and advice. This study highlights a significant need for improvement in the quality of service provided by pharmacy employees. Despite these challenges, the majority of consumers reported having a preferred pharmacy, and that employee knowledge is the most important criterion when choosing a pharmacy.

**Conclusions:**

Consumer distrust, in this context, is based on the incomplete knowledge or incompetency of pharmacy employees. Collective actions should be taken to improve the role of pharmacy employees and consequently improve the public trust in them, which can ensure better control of self-medication and reduce the instances of mistreatment.

**Supplementary Information:**

The online version contains supplementary material available at 10.1186/s12913-023-10471-1.

## Background

The prevalence of self-medication using over-the-counter (OTC) drugs is a growing worldwide concern [1–4]. This practice can lead to the irrational use of medicines and their related harmful effects, posing a significant problem for the healthcare system. Research has shown that more than half of the 266 examined drug side effects are related to OTC drugs [5]. Unfortunately, patients often rely on unreliable sources, such as the internet, advertisements, newspapers, family, and friends, for information about OTC drugs [6, 7]. Research conducted in Indonesia underscored the fact that when choosing drugs, consumers consider factors such as advertising, peer input, and the pharmaceutical company’s reputation [6]. While modern consumers often rely on the internet to address their healthcare concerns, research has shown that internet information regarding the choice of drugs can negatively affect treatment outcomes [8, 9]. This overreliance on nonprofessional sources can lead to the nonrational use of medicines, which is a concerning concept.

To address this issue, improving the relationship between patients and healthcare professionals can have a positive impact by reducing consumers’ loyalty to nonprofessional sources [10]. In developing countries, consumers are often unaware of the risks associated with self-medication and pharmaceutical side effects. Therefore, educating the public, increasing awareness about the hazards of self-medication, and strengthening advertising regulations are essential. Governments and healthcare organizations should promote rational and responsible self-medication practices [11–13]. Consumers should understand that patient-pharmacist and patient‒doctor relationships are crucial, and the information received from healthcare professionals cannot be replaced by that received from other sources [14]. By educating consumers and increasing their awareness of the risks of self-medication, healthcare professionals can play a vital role in promoting the safe and responsible use of OTC drugs.

Since patients can choose OTC drugs for themselves from pharmacies, it becomes necessary to provide them with proper pharmaceutical care [13–15]. Pharmaceutical care is a practice in which the practitioner takes responsibility for a patient’s drug-related needs and is held accountable for this commitment. During this practice, responsible drug therapy is provided to achieve positive patient outcomes. Studies have demonstrated that pharmaceutical care interventions, including medication dispensing, drug information provisioning, patient counselling, drug monitoring, adverse drug reaction monitoring, medication reconciliation, and drug protocol development, can reduce the occurrence of medication errors, improve adherence, and enhance patient education. Additionally, pharmaceutical care services can lead to a reduction in hospital admissions and mortality rates, which can contribute to improved healthcare efficiency. Promoting the role of pharmacists in providing pharmaceutical care services is critical for optimizing patient outcomes and reducing healthcare costs [16, 17].

Pharmacy employees play a crucial role in the healthcare system by providing primary healthcare services and comprehensive drug counselling to communities. They are often the first point of contact for patients seeking treatment for their symptoms, particularly in developing countries [18–21]. However, despite the potential of pharmaceutical care to improve patient outcomes, its implementation in practice is hindered by various obstacles, including a lack of resources, inadequate training, and regulatory barriers. Studies conducted in developing countries have shown a close relationship among patients’ choice of pharmacy, pharmaceutical care, and patient loyalty. A consumer’s decision when choosing a pharmacy and their level of satisfaction with the services offered can be influenced by the consistent application of pharmaceutical care. Therefore, overcoming the barriers to implementing pharmaceutical care services and promoting the role of pharmacy employees as reliable sources of healthcare services to improve patient outcomes and satisfaction are essential [22–24]. A comparative study between Poland and the United Kingdom (UK) found that Polish consumers prioritize low drug prices, while UK consumers value the knowledge and care provided by pharmacy employees. In addition, a larger percentage of Polish consumers exhibit a preference and trust towards specific pharmacies, thus indicating a need for improved pharmaceutical care in Poland [25].

Pharmaceutical care services as provided by community pharmacists have gained significant importance and are integral components of healthcare systems in developed nations. For instance, in Europe, the expansion and adoption of pharmaceutical care services as offered by community pharmacists have been observed in several countries [26]. Denmark, in particular, stands out for its nationwide consistency in the provision of these services [27]. In China, the initiative to integrate professional pharmacists into the community pharmacist role began in 2009, representing a pivotal change in the healthcare system that was aimed at enhancing the level of primary care services [28]. Conversely, in less developed countries, pharmaceutical care services provided by community pharmacies remain primarily focused on product-oriented practices [29]. There is a lack of data on the consumer perceptions of pharmaceutical care in Armenia, as no previous study has been conducted on this topic. In many countries, including Armenia, there is an increasing trend towards greater availability of OTC drugs, which highlights the need to develop pharmaceutical care as an aspect of modern primary care. However, pharmaceutical care provided by pharmacy employees is currently not standardized, thus it varies by the quality of recommendations, of information gathering, and of counselling practices. There are no specific professional requirements for pharmacy employees or for the pharmaceutical consultations they provide in the Republic of Armenia. This role is defined only by the “licensing” law, where the only requirement necessary for working in a pharmacy is to have a certificate certifying the professional education of a pharmacist/pharmacy technician. At the same time, the law lacks a concept and a normative-legal definition for the term “pharmaceutical care”; therefore, there are also no standards for the implementation of such care, particularly regarding the advisory function performed by a pharmacy employee when dispensing OTC drugs as an aspect of responsible self-medication.

Armenia’s evolving healthcare system, which is marked by limited access to health insurance and pharmaceutical services, is the context for this study. The specific dynamics within Armenia may yield unique consumer perceptions of pharmaceutical care when compared to well-established healthcare systems. Amid diverse global healthcare landscapes, countries undergoing transitions or lacking in universal health insurance face distinctive challenges. Our findings in Armenia contribute to a broader understanding of pharmaceutical care within varying healthcare contexts, offering insights for regions under different levels of healthcare development. Our study bridges the gap in the understanding of pharmaceutical care perceptions in Armenia’s transitional healthcare system. It provides crucial insights into consumer concerns, expectations, and preferences, offering guidance for healthcare stakeholders in Armenia and similar regions. This research supports the development of tailored healthcare strategies. In summary, the contribution of our research’ lies in the enrichment of global knowledge by illuminating pharmaceutical care perceptions in a dynamic healthcare context. This work has the potential to enhance healthcare policies and practices, not only in Armenia but also in other countries facing analogous healthcare challenges.

The primary goal was to evaluate consumer self-medication practices, assess the pharmaceutical care offered by pharmacy employees and analyse the level of consumer satisfaction with this care. Additionally, the research was aimed at investigating the main criteria deemed important by consumers when selecting a pharmacy within Armenia.

## Methods

### Study design and sample

The study was aimed at providing a comprehensive assessment of the pharmaceutical care services and consumer choices in selecting a pharmacy by investigating two distinct groups: pharmacy consumers and pharmacy employees. To gather multifaceted data on this topic, an anonymous questionnaire-based cross-sectional design [30] was employed. The data collection instruments were developed by utilizing standard WHO (World Health Organization) survey questionnaires [31] based on a comprehensive literature review [32–37]. Following survey development, a pilot study was meticulously conducted to evaluate the survey’s performance, enhance the research procedures, and optimize the data collection efficiency. The pilot study closely emulated the main study in terms of its methodology and sample characteristics, offering a robust platform for assessment. The feedback obtained during the pilot study phase played a pivotal role in refining the survey instruments to ensure their clarity, relevance, and overall comprehensibility. To assess the internal consistency and reliability of the survey instruments, we computed Cronbach’s alpha [38]. This statistical measure substantiated the robustness of our data collection instruments by confirming their capacity to consistently and reliably measure the constructs used in the study.

The study involved surveying 1308 pharmacy consumers residing in the regions of Armenia and its capital city of Yerevan, and 597 pharmacy employees in Yerevan. The questionnaires for both groups were divided into several sections and contained open- and closed-ended questions.

The questionnaires for pharmacy consumers were aimed at collecting information about the demographic characteristics of the participants, their interactions with pharmacy staff, their sources of information regarding medications and consumer opinions on the quality of the counselling provided in community pharmacies.

On the other hand, the questionnaires for pharmacy employees were employed under the following objectives:

- To gather general information about the participants’ professional background and experience in the field.
- To assess the knowledge of pharmacy staff regarding minor ailments and OTC drugs.
- To evaluate the quality of pharmaceutical care provided to consumers by pharmacy staff.

The sample sizes for the research on consumers and pharmacy employees were calculated using the sample size formula, which has a degree of reliability of 97% and a permissible error rate of 3%. A sample size of 597 employees was calculated for the population of pharmacy employees in Yerevan based on the total number of 1096 pharmacies, and a sample size of 1308 was calculated for the population of Armenia based on a population size of 2,972,700.

### Ethical approval and consideration

The Ethics Committee of Yerevan State Medical University approved the questionnaires during a routine session (Protocol no. 10, dated on 02.02.2020). The survey was conducted with the consent of the participants, and their confidentiality was ensured. Participants were notified that the data they provided would be used and published. To preserve the ethical side of the work and motivate participants to complete the questionnaires, the scientific value and objectives of the study and clear instructions for completing the questionnaire were provided to each participant.

### Data collection

The study was carried out between March 2020 and November 2021. Four pharmacists supervised the completion of the questionnaires, for which they were provided training beforehand.

The study was conducted in a community pharmacy. To ensure the reliability and impartiality of the data, an Excel random number generator was used to select participants. The random assignment of participants is a widely used method in research studies for reducing the impact of confounding variables and minimizing bias in the study results.

### Data analysis

The data from the questionnaires were entered into a custom-designed database using the Statistical Package for Social Sciences (SPSS, version 23.0) software. This program enables an analysis of the survey data and presents results through the use of frequency and percentage statistics. The impact of the educational levels of pharmacy employees on the quality of the pharmaceutical care they provide was evaluated. Statistical significance was set at *p* < 0.05.

## Results

The study began with 597 pharmacy employees completing the survey during an 8-week study period. It took participants 12–15 min each to complete the questionnaire. According to the results of the research, 59.9% of the participating employees were pharmacy technicians by profession, and 21.9% had higher pharmaceutical education. Moreover, 17% of the participants were classified as students who had not attained a professional qualification at the time of the study (see Table 1).

**Table 1:** Demographics of pharmacy employees

**Variable**	**Frequency (%)**	
Age (*N* = 591)	≤ 24	192 (32.1)
	25–35	121 (20.2)
	36–45	191 (31.9)
	⩾ 46	87 (14.5)
Sex (*N* = 591)	Female	491 (82.2)
	Male	100 (16.7)
Level of education (*N* = 591)	Pharmacist	131 (21.9)
	Pharmacy technician	358 (59.9)
	Student	102 (17.0)
Years of practice (*N* = 591)	≤ 1 year	161 (26.9)
	2–3 years	191 (31.9)
	4–7 years	137 (22.9)
	8–10 years	54 (9.0)
	≥ 11 years	54 (9.0)

Response rate = 99%

*N* The number of respondents completing the item

The study results suggest that a significant proportion of pharmacy employees frequently dispense OTC drugs on their basis of their own advice. Specifically, 21.9% of pharmacy employees dispense OTC drugs 11–15 times a day, while 24.1% dispense them 16–20 times a day (Table 2). The table illustrates the association between pharmacy employee educational levels and the quality of pharmaceutical care provided. A highly significant association was observed, as indicated by a *p* value of 0.000.

**Table 2:** Pharmaceutical care provided by pharmacy employees

Variable	**Frequency (%)**	**Impact of educational levels on pharmaceutical care quality**			***P* value**
		**Pharmacists (%)**	**Pharmacy technicians (%)**	**Students (%)**	
How many times a day do you dispense over-the-counter medications on your own advice? (*N* = 591)					0.000
Up to 5 times	114 (19.0)	27 (20.6)	49 (13.7)	35 (34,3)	
6–10 times	125 (20.9)	31 (23.6)	97 (27.1)	31 (30.4)	
11–15 times	131 (21.9)	22 (16.7)	93 (26)	14 (13.7)	
16–20 times	143 (24.1)	27 (20.6)	69 (19.3)	21 (20.6)	
21–25 times	30 (5.0)	7 (5.3)	22 (6.1)	0	
More than 26 times	48 (8.0)	17 (12.9)	28 (7.8)	1(1)	
Do you inform the consumer about medicine instructions before dispensing medicines? (*N* = 591)					0.513
Yes	263 (44.0)	86 (65.6)	206(57.5)	62 (60.8)	
No	233 (39.0)	27 (20.6)	95 (26.5)	25 (24.5)	
Sometimes	95 (15.9)	18 (13.7)	57 (15.9)	15 (14.7)	
Do you warn the consumer about the possible side effects before dispensing medicines?					0.039
Yes	155 (25.9)	42 (32)	82(22.9)	29 (28.4)	
No	251 (42.0)	56(42.7)	161(45)	37 (36.3)	
Sometimes	185 (30.9	33(25.1)	115(32.1)	36 (35.3)	

Response rate = 99%

*N* The number of respondents completing the item

Furthermore, the quality of the pharmaceutical care provided by pharmacy employees was evaluated. The findings reveal a need for improvement in this area. In particular, 44% of pharmacy employees explicitly indicated engaging in a consistent practice of informing consumers about medication instructions. In contrast, 15.9% of them provide such information only intermittently, and a notable 39% refrain from informing consumers altogether. This indicates that a considerable proportion of pharmacy employees fail to provide adequate information to consumers regarding medication instructions. The variable did not exhibit a significant association among the different sample groups (*p* = 0.513), indicating that educational level does not significantly impact the practice of providing medicine instructions to consumers.

Similarly, the study found that only 25.9% of pharmacy employees clearly stated that they inform consumers about the potential side effects of medicines before dispensing them. Furthermore, 30.9% of them occasionally provide information to consumers, while 42% of pharmacy employees omit discussing potential side effects entirely. These findings suggest a need for greater emphasis on providing comprehensive information to consumers about medication instructions and potential side effects. A highly significant association (*p* = 0.039) was found. Pharmacy employees with different educational levels significantly differ in their practice of advising consumers about possible side effects.

The next stage of the study involved the participation of 1308 pharmacy consumers. Their characteristics are summarized in Table 3. The majority of participants were in the 51 to 60 age group, with the lowest level of involvement of those aged 18 to 29. Among the participants, 57.9% were from the capital and 39.5% were from rural areas. The majority of participants (38.7%) held postgraduate degrees.

**Table 3:** Demographics of pharmacy consumers (*n* = 1275)

**Variable**	**Frequency (%)**	
Age (years) (*N* = 1275)	18–29	209 (15.9)
	30–40	229 (17.5)
	41–50	246 (18.8)
	51–60	310 (23.7)
	⩾61	281 (21.4)
Level of education (*N* = 1275)	None	0
	High school	332 (25.3%)
	Postgraduate degree	507 (38.7%)
Place of Residence (*N* = 1275)	Undergraduate degree	436 (33.3%)
	Central city	758 (57.9%)
	Rural areas	517 (39.5%)

Response rate = 97.5%

*N* The number of respondents completing the item

With the daily increase in the availability of OTC drugs and, therefore, the prevalence of self-medication, it is extremely important for patients to use only reliable and professional sources of consultation, which, according to research, has a positive effect on the patient’s treatment. In this regard, the survey revealed that most consumers use medicines independently of consultation and generally rely on personal experiences (29.2%), 15.5% use medicines according to pharmacy employee consultation, 25% visit a physician, 8.8% ask for friends’/neighbours’ help, 10.7% use the internet, and 8% rely on advertising (Table 4). The data show that 56.7% of respondents take medication without consulting healthcare specialists.

**Table 4:** Sources of counselling

**Variable**	**Frequency (%)**	
Sources of counselling (*N* = 1275)	Personal experiences	383(29.2)
	Friends/neighbours’ experiences	116 (8.8)
	Internet information	141 (10.7)
	Advertisement information	105 (8.0)
	A physician	327 (25.0)
	A pharmacy employee	203 (15.5)

Response rate = 97.5%

*N* The number of respondents completing the item

To make the research results more comprehensive, we also analysed the reasons why consumers self-medicate without consulting a pharmacy employee. The respondents clarified that 34.5% of consumers never agree to buy drugs on the advice of a pharmacy employee, and 29.9% show an inconsistent position by responding that they only sometimes agree to buy (Table 5). For consumers, the main reasons for such behaviour were reported as follows: 32% based this on their previous unsuccessful experience, 41.8% did not trust the pharmacy employee, 15% cited the pharmacological company that makes the recommended drug, and 11% cited the price of the drug. Irrational use of drugs by consumers and many health problems occur as a result.

**Table 5:** Attitudes regarding pharmacist competence

**Variable**	**Frequency (%)**	
Have you ever refused to purchase a drug that was recommended by a pharmacist? (*N* = 1275)	Yes	452 (34.5)
	No	431 (32.9)
	Sometimes	392 (29.9)
If yes, what were the main reasons for such behaviour? (*N* = 452)	Previous bad experience	145 (32.0)
	Lack of trust in the pharmacy employee	189 (41.8)
	Medicine prices	50 (11.0)
	Lack of trust in the pharmaceutical company	68 (15.04)
Can a pharmacy employee answer your questions fully? (*N* = 1275)	Yes	314 (24.0)
	No	340 (25.9)
	Sometimes	621(47.4)
Does the pharmacy employee ask you questions to understand your health condition before recommending medicine? (*N* = 1308)	Yes	366 (27.9)
	No	248 (18.9)
	Sometimes	157 (12.0)
	It depends on the pharmacy employee	138 (10.5)
	It is difficult for me to remember	366 (27.9)
How would you evaluate the services provided by pharmacy employees on a point system (very bad: 1 point, bad: 2 points, enough: 3 points, fine: 4 points, very good: 5 points)? (*N* = 1308)	1	55 (4.2)
	2	526 (40.2)
	3	385 (29.4)
	4	179 (13.7)
	5	130 (10)

Response rate = 97.5%

*N* The number of respondents completing the item

For the question “Can a pharmacy employee answer your questions fully?” Most consumers (47.4%) answered that they were sometimes satisfied with the answers of a pharmacy employee, 24% of them clearly stated that a pharmacy employee was aware of the answers of their questions, and 25.9% said that a pharmacist could not answer their questions.

During the survey, we probed more deeply into this with the question: “Does the pharmacy employee question you to understand your health condition before recommending medicine?” The relative majority of consumers answered yes, and the following response rates were obtained: yes – 27.9%, no – 18.9%, sometimes - 12%, depends on the pharmacy employee - 10.5%, and I find it difficult to remember - 27.9%. Complaints are the main source of feedback for pharmacy employees regarding their performance in medicine discharge, as well as in the detection of dangerous symptoms, so the interest of pharmacy employees in complaints should be high.

The study investigated the quality of services provided by pharmacy employees using a 5-point evaluation scale with response options that ranged from 1 (very bad) to 5 (very good) in assessing customer satisfaction. The scale was designed to enable easy identification of positive and negative results. The findings showed that a significant proportion of consumers rated the service provided by pharmacy employees as poor (40.2%), while 29.4% rated it as satisfactory, and only 4.2% rated it as very poor. These results suggest that there is room for improvement in the quality of service provided by pharmacy employees.

The next stage of the sociological survey involved determining whether consumers have a preferred pharmacy that they use consistently. The results show that most consumers (59.9%) have a preferred pharmacy, and only 37.5% of respondents do not (Table 6). This indicates public trust in some pharmacies and pharmacy employees.

**Table 6:** Do you have a preferred pharmacy where you always shop? (*N* = 1308)

**Variable**	**Frequency (%)**
Yes	784 (59.9)
No	491 (37․5)

Response rate = 97.5%

*N* The number of respondents completing item

The main criteria for choosing a preferred pharmacy can be seen in Table 5.

The main criterion determined was the knowledge of pharmacy employees (42%). Some other relevant factors included pharmacy location (15%), product assortment (17%), price of medicines (16%), and the level of care provided by pharmacy employees (10%) (Table 7).

**Table 7:** Factors influencing the choice of a particular pharmacy (*N* = 654)

**Variable**	**Frequency (%)**
Knowledge of pharmacy employee	278 (42%)
Product assortment	111 (17%)
Price of medicines	105 (16%)
Pharmacy location	95 (15%)
Care provided by pharmacy employees	65 (10%)

Response rate = 97.5%

*N* The number of respondents completing the item

## Discussion

This study is the first in the Republic of Armenia to investigate pharmaceutical care services and consumers’ choice of pharmacy. Providing proper pharmaceutical care is a crucial aspect of healthcare, while the study reveals that many residents in Armenia engage in self-medication without consulting professional sources. While the WHO encourages self-medication for certain minor illnesses under the guidance of a pharmacist, the survey findings suggest that most consumers do not seek consultation or other services provided by pharmacies and rather make their own decisions about medication use. They report often relying on information from friends, neighbours, or the internet, which may not always be reliable or accurate. This can lead to potential risks and result in dangerous consequences for consumers. The study’s findings are consistent with those of similar surveys conducted in Singapore and China, where the majority of participants who self-medicated did not seek the advice of a pharmacist and only consulted a doctor or pharmacist after determining that self-medication was ineffective. They self-medicated based on their previous experience or on the advice of friends [34, 35].

The growing practice of self-medicating with OTC drugs results in the mistrust towards pharmacists [36]. The study findings indicate that consumers’ previous negative experiences have contributed to their mistrust of pharmacy employees, primarily due to the inability to obtain proper advice from pharmacy staff. According to a study, most pharmacy employees show a lack of interest in the patient’s complaint and fail to provide adequate information on the possible side effects of dispensed drugs. In addition, a significant proportion of pharmacy employees do not provide instructions on the proper use of the drugs. Only a small percentage of consumers (24%) expressed satisfaction with the answers provided by pharmacy employees. This practice runs contrary to the concept of pharmaceutical care, which emphasizes the importance of understanding the nature of the complaint, confirming or denying the patient’s self-diagnosis, and providing appropriate advice. Incomplete advice received from pharmacy employees leads to a lack of trust in their advice. In contrast, European countries have recognized that the provision of drug advice is an important factor in building trust between consumers and pharmacy employees. Our study found that the majority of consumers in the Republic of Armenia do not have confidence in the services provided by pharmacy employees and are generally dissatisfied with the quality of those services. More than one-third of consumers (40.2%) rated the service provided by pharmacy employees as poor, indicating a significant need for improvement. Another 29.4% of consumers rated the service as satisfactory, suggesting that there are still areas in need of improvement. Other studies have shown that the higher the frequency of consultation and monitoring is, the higher the level of consumer satisfaction with the services provided by the pharmacy employee [24, 25]. Our study’s results are consistent with those of similar studies conducted in Germany, which also found that pharmacy employees provided low-quality consultation, with dosage information being the most commonly provided information and information on side effects being the least commonly provided. Similarly, in Qatar, a study showed that only 37% of consumers felt that pharmacy staff were knowledgeable and ready to answer all questions [37, 38].

The insufficient knowledge and inadequate communication skills of pharmacy staff may contribute to the limited communication with patients and hinder the formation of trust in relationships [39–41]. According to our research, pharmacies have an exceedingly minor percentage of employees possessing higher education (pharmacists)․ This is due in part to the low salaries and limited opportunities for advancement within the pharmacy sector. As a result, many highly educated pharmaceutical specialists prefer to seek employment in other sectors. Notably, there is currently no legal requirement in regulatory affairs (RA) for pharmacies to employ at least one specialist with higher education. This lack of regulation is a major concern, as the knowledge and expertise of pharmacists play a critical role in ensuring the safe and effective use of medications. The research has shown that some pharmacies employ students in this role. This highlights a discrepancy between the requirements set out in the Pharmacy Licensing Act and the actual staffing practices followed in many pharmacies. Importantly, the professional staff of a pharmacy should only include individuals with full pharmaceutical education, such as pharmacists and pharmacy technicians [42, 43].

Nevertheless, the survey we carried out in Armenia shows that the majority of consumers have a preferred pharmacy. It was interesting to determine what criteria consumers use in choosing a pharmacy. When choosing a pharmacy, the most important criterion for consumers was the knowledge of the employees. Importantly, the price of drugs was not a major criterion for consumers and ranked only third among the overall criteria. One could posit that a significant portion of consumers place a higher value on receiving valuable advice than they do on the price factor, thus displaying a readiness to pay a premium for such valuable guidance. These results can be explained by the fact that counselling is a compulsory part of dispensing. In this regard, Armenian pharmacists need to exert greater efforts toward promoting these endeavours. Studies in the USA show that patients are even willing to pay extra and spend more time at the pharmacy to obtain pharmacist advice [44–47]. The opposite picture was obtained during a study in Malaysia, where the most important indicators were shown to be the location of the pharmacy, the assortment of products, and the prices, while the knowledge of the pharmacy staff and good service were not as important to consumers. This may be a result of consumers being unaware of the role and responsibilities of pharmacy employees in the healthcare system. Such consumers do not deem the pharmacist to be the most reliable source provider for OTC drugs [34].

### Limitations of the study

The data collection for the study was short-term and ad hoc, and no studies were conducted over time to determine if consumer preferences and responses have changed as a result of any potential additional factors. Further study is recommended to be repeated in a year. An extended-duration study would offer a clearer understanding of this topic of interest.

## Conclusions

Based on the research data, it can be concluded that in Armenia:

- Self-medication without pharmacist consultation is quite common.
- Pharmacy employees provide inadequate therapy for simulated minor ailments.
- Most consumers do not trust and are unsatisfied with the pharmacy staff’s guidance regarding OTC drugs.

However, during the survey, consumers reported considering the knowledgeability of the pharmacy employee to be very important when choosing a pharmacy. In other words, consumer distrust is based on the insufficient knowledge of pharmacy employees. Collaboration between pharmacists, pharmacies, and the government is needed to increase the role of pharmacists and the public trust in them, which would enable better control of the self-medication process and reduce the number of errors made.

There is a need to improve the professional behaviour of Armenian pharmacy employees regarding pharmaceutical care. The allocation of resources for the formulation of algorithms and protocols aimed at addressing minor ailments is shown to be important. Such measures are designed to guide the professional conduct of pharmacists, thus empowering their active involvement in the sphere of primary healthcare provision.

### Supplementary Information

**Additional file 1.** Pharmacy consumer questionnaire.

**Additional file 2.** Pharmacy employee questionnaire.

## Data Availability

The datasets generated during and (or) analysed during the current study are available from the corresponding author upon reasonable request.

## Abbreviations

RA: Republic of Armenia
OTC: Over-the-counter
UK: United Kingdom
WHO: World Health Organization
SPSS: Statistical Package for the Social Science
