# Supplementary material for: Evaluating consumer self-medication practices, pharmaceutical care services, and pharmacy selection: a quantitative study
Source: BMC Health Serv Res. 2024 Jan 3;24:10. doi: 10.1186/s12913-023-10471-1 (PMC10765736; doi:10.1186/s12913-023-10471-1)
Supplement: Supplementary file 1 — Additional file 1. Pharmacy consumer questionnaire. [file 12913_2023_10471_MOESM1_ESM.docx]

**QUESTIONNAIRE**

Pharmacy consumer questionnaire

SECTION 1: DEMOGRAPHIC CHARACTERISTIC

1. Age

- 18-29
- 30-40
- 41-50
- 51-60
- ⩾61

1. Level of education

- None
- High school
- Postgraduate degree
- Undergraduate degree

1. Place of Residence

- Central city
- Rural areas

4. Have you been diagnosed with any health problems by a doctor?

- Yes
- No

5. If yes, what kind?

- Cardiovascular
- Gastrointestinal
- Diseases of the nervous system
- Internal diseases (diabetes, cancer)
- Oncological
- Other
- I don't have any health problems

SECTION 2: CONSUMER INTERACTIONS WITH PHARMACY EMPLOYEES AND THEIR SOURCES OF INFORMATION REGARDING MEDICINES.

6. How many times have you consulted a doctor in the last month?

- I did not apply
- Once
- Twice
- Three or more times

7. How many times have you shopped at the pharmacy in the last month?

- I didn't do it
- Once
- Twice
- Three or more times

1. Have you ever refused to purchase a drug that was recommended by a pharmacist?

- Yes
- No
- Sometimes

1. If yes, what were the main reasons for such behaviour?

- Previous bad experience
- Lack of trust in the pharmacy employee
- Medicine prices
- Lack of trust in the pharmaceutical company

1. Where do you typically seek information or advice when purchasing medication?

- Personal experiences
- Friends/neighbours' experiences
- Internet information
- Advertisement information
- A physician
- A pharmacy employee

SECTION 3: CONSUMER OPINIONS ON THE QUALITY OF THE COUNSELLING PROVIDED IN COMMUNITY PHARMACIES.

1. Does the pharmacy employee ask you questions to understand your health condition before recommending medicine?

- Yes
- No
- Sometimes
- It depends on the pharmacy employee
- It is difficult for me to remember

1. Can a pharmacy employee answer your questions fully?

- Yes
- No
- Sometimes

1. Does the pharmacy employee later inquire how the drugs purchased last time helped?

- Yes
- Sometimes
- No

1. Do the pharmacy employees meet your expectations in terms of the quality of the drugs they recommend?

- Yes
- No
- Sometimes

1. Do you trust the advice of the pharmacy employee?

- Yes
- No
- Sometimes

1. In the case of minor ailments (for example, diarrhea), when buying a drug based on the advice of a pharmacy employee, does the pharmacy employee warn about the possible side effects of the recommended drug?

- Yes
- No
- Sometimes
- Depends on the employee of the given pharmacy
- I have trouble remembering

1. In case of minor ailments (for example, diarrhea), when buying medicine on the advice of a pharmacy employee, does the pharmacy employee warns about the order of use of the medicine they recommend?

- Yes
- No
- Sometimes
- Depends on the employee of the given pharmacy
- I have trouble remembering

1. In case of minor ailments (for example, diarrhea), have you ever consulted a doctor based on the advice of a pharmacy employee?

- Yes
- No
- Sometimes
- I have trouble remembering

1. In case of minor ailments (for example, diarrhea), are you interested in the pharmacological group of drugs the pharmacy employee offers?

- Always
- Sometimes
- No

1. How long do you think it takes to make a preferred purchase at the pharmacy?

- up to 5 minutes
- up to 10 minutes
- up to 20 minutes
- time is not important, the important thing is to get answers to pressing questions

1. Has a pharmacy employee ever contacted your doctor to clarify any questions?

- Yes
- No

1. How would you evaluate the services provided by pharmacy employees on a point system (very bad: 1 point, bad: 2 points, enough: 3 points, fine: 4 points, very good: 5 points)?

- 1
- 2
- 3
- 4
- 5

1. Using the scoring system, how would you rate the knowledge of the employee at the pharmacy you regularly visit on a scale of 1 to 5? (very bad: 1 point, bad: 2 points, enough: 3 points, fine: 4 points, very good: 5 points)?

- 1
- 2
- 3
- 4
- 5

1. Do you have a preferred pharmacy where you always shop?

- Yes
- No

1. If yes, please mention factors influencing the choice of a particular pharmacy

- Knowledge of pharmacy employee
- Product assortment
- Price of medicines
- Pharmacy location
- Care provided by pharmacy employees

THANK YOU FOR PARTICIPATING!
