# Supplementary material for: Evaluating consumer self-medication practices, pharmaceutical care services, and pharmacy selection: a quantitative study
Source: BMC Health Serv Res. 2024 Jan 3;24:10. doi: 10.1186/s12913-023-10471-1 (PMC10765736; doi:10.1186/s12913-023-10471-1)
Supplement: Supplementary file 2 — Additional file 2. Pharmacy employee questionnaire. [file 12913_2023_10471_MOESM2_ESM.docx]

**QUESTIONNAIRE**

Pharmacy employee questionnaire

SECTION 1: DEMOGRAPHIC CHARACTERISTIC

1. Age

- ≤ 24
- 25-35
- 36-45
- ⩾ 46

1. Sex

- Female
- Male

1. Level of education

- Pharmacists
- Pharmacy technician
- Student

1. How many times a day do you dispense over-the-counter medications on your own advice?

- up to 5 times
- 6-10 times
- 11-15 times
- 16-20 times
- 21-25 times
- More than 26 times

SECTION 2: ASSESS PHARMACY EMPLOYEES KNOWLEDGE OF MINOR AILMENTS, WITH EXAMPLES SUCH AS DIARRHEA, AND OVER-THE-COUNTER (OTC) MEDICATIONS.

1. What are common causes of diarrhea?

……………………………………………………….

1. What are red flags or severe symptoms of diarrhea that warrant a visit to the doctor?

……………………………………………………….

1. What are the top-selling drugs for diarrhea according to customer demand? Please specify the first 5 most common ones.

……………………………………………………...

1. Your most recommended medicines for diarrhea. Please specify the first 5 most common ones.

………………………………………………………

1. According to you, what are the main side effects of the drugs you recommend for diarrhea? Please specify the first 5 most common ones.

…………………………………………………......

SECTION 3: Evaluation of the quality of pharmaceutical care provided to consumers by pharmacy staff.

1. In the case of which complaints do patients use the advice of a pharmacy employee the most?

- Pain syndrome (different types of pain)
- Cold, cough
- Gastrointestinal tract disorders (drowsiness, heartburn, diarrhea, constipation)
- Nervous problems

1. Do you inform the consumer about medicine instructions before dispensing medicines?

- Yes
- No
- Sometimes

1. Do you warn the consumer about the possible side effects before dispensing medicines?

- Yes
- No
- Sometimes

1. On what principle do you offer the medicines?

- Accessibility
- Quality
- Producing organization
- Doctor's appointment
- Your option

1. To get information about over-the-counter drugs, you use:

- From the Internet
- From standard schemes
- From media, TV, newspaper
- From professional books
- From lectures
- From the tab sheet
- Your option

1. Do you have a standard scheme according to which you ask the questions to the consumer in order to decide what medicine to recommend?

- Yes
- No
- Depends on the case

1. Would you like to have an approved standard scheme, according to which you ask the consumer the questions in that order, in order to decide what medicine to recommend?

- Yes
- No
- Depends on the case

1. What advantages would the standard scheme bring according to you?

- Security
- Less responsibility
- Protection
- Possibility of quick orientation
- Your option

THANK YOU FOR PARTICIPATING!
